# RNAi-mediated knockdown of arginine kinase genes leads to high mortality and negatively affect reproduction and blood-feeding behavior of *Culex pipiens pallens*

**DOI:** 10.1371/journal.pntd.0010954

**Published:** 2022-11-22

**Authors:** Kun Qian, Qingqing Guan, Haoyu Zhang, Nan Zhang, Xiangkun Meng, Hongxia Liu, Jianjun Wang

**Affiliations:** 1 College of Plant Protection, Yangzhou University, Yangzhou, China; 2 Department of Infectious Disease Control, Shanghai Municipal Center for Disease Control and Prevention, Shanghai, China; Creighton University, UNITED STATES

## Abstract

**Background:**

Arginine kinase (AK) is one of the crucial enzymes involved in energy metabolism in invertebrates, and has been proposed as the target for RNA interference (RNAi)-based control of agricultural insect pests. While there is only one AK gene in most insects, two AK genes were identified in *Culex pipiens pallens*, the primary vector of lymphatic filariasis and epidemic encephalitis.

**Methods:**

The full-length cDNA sequences of *CpAK1* and *CpAK2* genes were obtained by reverse transcription PCR(RT-PCR) and rapid amplification of cDNA ends (RACE). The expression levels of *CpAK1* and *CpAK2* in different developmental stages and tissues were detected by reverse transcription quantitative PCR (RT-qPCR). The role of *CpAK1* and *CpAK2* in the reproduction and blood feeding behavior was analyzed using RNA interference (RNAi).

**Results:**

Full-length cDNAs of *CpAK1* and *CpAK2* were isolated from *Cx*. *pipiens pallens*. Analysis of the expression pattern revealed that the mRNA level of *CpAK1* was significantly higher than *CpAK2* in all development stages and tissues examined, and the expressions of both *CpAK1* and *CpAK2* were upregulated in response to blood feeding. The co-knockdown of *CpAK1* and *CpAK2* mediated by RNAi led to high mortality (74.3%) of adult female mosquitoes and decreased hatchability (59.9%). Remarkably, the blood feeding rate and the engorgement rate of the female mosquitoes were negatively affected by co-injection of dsRNAs targeting *CpAK1* and *CpAK2*.

**Conclusion:**

*CpAK1* and *CpAK2* were detected in all developmental stages and tissues, but showed divergence in expression level. RNAi-mediated knockdown of AK genes leads to high mortality and negatively affect blood-feeding behavior of *Cx*. *pipiens pallens*, suggesting that *AK* could be used for the target of RNAi-based mosquito control in the future.

## Author summary

The rapid expansion of insecticide resistance in mosquito populations prompted the development of novel control strategies, such as RNA interference (RNAi). As the only member of the phosphagen kinase family in insects, arginine kinase (AK) plays an important role in insect energy metabolism, however, characterization of mosquito AK is still limited. We cloned the full-length cDNAs encoding *CpAK1* and *CpAK2* from *Cx*. *pipiens pallens*, and investigated their spatial-temporal expression patterns as well as the effects of RNAi-mediated knockdown of *CpAK1* and *CpAK2* on mosquito reproduction and blood-feeding behavior. We found that the expression levels of both *CpAK1* and *CpAK2* were upregulated in response to blood feeding, and knockdown *CpAKs* led to high mortalities of adult female mosquitoes and decreased hatchabilities. The blood feeding rate and the engorgement rate of the female mosquitoes were negatively affected by co-injection of dsRNAs targeting *CpAK1* and *CpAK2*. The findings of this study indicate that technology based on RNAi of AK might provide a new way to control mosquito vectors.

## Introduction

Mosquitoes are vectors of a variety of viral pathogens causing infectious diseases that adversely affect human health. To eliminate the deadly mosquito-borne diseases such as dengue, chikungunya, yellow fever, and Zika, chemical insecticides have been widely used in public health to control mosquitoes. However, excessive use of insecticides caused mosquitoes to develop resistance to almost all known classes of insecticides including organochlorines, organophosphates, carbamates and pyrethroids, leading to a number of outbreaks of mosquito-related diseases in recent years [1,2]. To adequately manage insecticide resistance, there is an urgent need to develop new control strategies that will be effective in the light of existing resistance.

Arginine kinase (E.C.2.7.3.3; AK) is a kind of phosphagen kinases widely distributed in invertebrates such as nematodes, arthropods and mollusks [3]. AK is homologous to creatine kinase (CK) in vertebrates and acts as ATP-buffering system by catalyzing the reversible reaction between arginine and ATP [3]. In addition to its role in invertebrate energy metabolism, there are increasing evidences suggesting that AK is involved in development, stress response and immune resistance [4]. Particularly, RNA interference (RNAi)-mediated knockdown of insect AKs led to retarded development and increased mortality [5–8],indicating that AK was a potential target for the development of highly selective insecticides and RNAi-based control of insect pests.

As an opportunistic mammalian blood feeder, *Culex pipiens pallens* is the primary vector of lymphatic filariasis and St. Louis encephalitis [9–11]. In this study, two AK genes, *CpAK1* and *CpAK2*, were found in the genome of *Cx*. *pipiens pallens*. The full length cDNAs of *CpAK1* and *CpAK2* were cloned, and their spatial-temporal expression patterns were analyzed and compared. The roles of *CpAK1* and *CpAK2* in mosquito reproduction and blood-feeding behavior were also explored by RNAi. The results are of great scientific significance to further reveal the structure and function of insect AKs, as well as to develop new RNAi-based mosquito control strategy.

## Materials and methods

### Ethics statement

The animal experiments were conducted according to the guidelines of the Experimental Animal Ethics Committee of Yangzhou University (Approval no. DWLL-201912-001).

### Mosquito rearing

*Cx*. *pipiens pallen*s colony were maintained in the insectary at 26 ± 2°C and 75 ± 10% RH (relative humidity) under a 12:12h light:dark photoperiod. The larvae were reared in water supplemented with the nutritionally complete rat chow. Adult mosquitoes were kept in cages and fed with 5% sucrose water, and 3-day-old females were fed with fresh mouse blood to induce egg-laying.

### Total RNA isolation and reverse transcription

Total RNAs were extracted from the whole bodies of different developmental stages or specific tissues in female adults using the SV total RNA isolation system (Promega, Madison, WI). First-strand cDNA was synthesized from total RNA using the Primescript First-Strand cDNA Synthesis kit (TaKaRa, Dalian, China), according the manufacturers’ instructions. The development stages of the test mosquitoes used were as follows:1-day-old egg; 2-day-old larva; 5-day-old larva; 8-day-old larva; 11-day-old larva;1-day-old pupa; 1-day-old female adult and 3-day-old female adult. Total RNA was isolated from 1-day-old eggs (3 rafts), 2-day-old larvae (100 larvae), 5-day-old larvae (50 larvae), 8-day-old larvae (20 larvae), 11-day-old larvae (10 larvae), 1-day-old pupae (5 pupae), 1-day-old female adults (3 adults) and 3-day-old female adults (3 adults) for each biological replicate. The 3-day-old female adult mosquitoes without feeding blood were dissected after starvation for 24 hours, and their heads, salivary glands, thorax, midgut, Malpighian tube, ovaries and fat bodies were collected. The 5-day-old adult female mosquitoes after blood feeding were dissected, and the ovaries were collected. A total of 60 mosquitoes were dissected for each biological replicate.

### Polymerase chain reaction and rapid Amplification of cDNA Ends (RACE)

Based on the genomic sequence information of *Culex pipiens quinquefasciatus* (AK1 gene GenBank accession number EDS30648.1, AK2 gene GenBank accession number EDS33211.1), a closely related species of *Cx*. *pipiens pallens*, primers for amplifying AK gene fragments were designed by Primer Premier 5 software (Table 1). PCR reactions were performed with LA Taq DNA polymerase (TaKaRa, Dalian, China). In order to complete the cDNA sequences of *CpAK1* and *CpAK2*, 5’-RACE and 3’-RACE reactions were performed using the SMART RACE cDNA Amplification Kit (Clontech, Mountain View, CA, USA), according to the manufacturer’s instructions.

**Table 1:** The primers used in this study.

Description	Primer name	Sequence (5′ to 3′)
ORF amplification	*CpAK*1-F	ATGGGTGGTTGTGCGTCCAA
	*CpAK*1-R	TTACAGTGACTTCTCAATCTTGATGAGT
	*CpAK*1-5’RACE-in	TGTCCTTGGACGCACAACCACCCAT
	*CpAK*1-5’RACE-out	TTCGCCACTTCCGGCAGCTGTTTCG
	*CpAK*1-3’RACE-in	AACAAGCGTCGCATGGGTCTGACTG
	*CpAK*1-3’RACE-out	TGAAGGGCAAGTTCTACCCGCTGAC
	*CpAK*2-F	ATGGCTTCGCGGGAGGCGTT
	*CpAK*2-R	CTACACCTTTGGTGCCGAC
	*CpAK*2-5’RACE-in	TCTCAGTGACCTGCTCGTCCGTGGG
	*CpAK*2-5’RACE-out	TTCCGCAGCGAACCCTCGTCGAAAC
	*CpAK*2-3’RACE-in	AGGAAGAGGAGAAACCAGCGGAAGC
	*CpAK*2-3’RACE-out	CCGTGGAGACCATCGGCAAAGACAT
qRT-PCR	*CpAK1*-qF	GCCAACGCTTGCCGTTACTG
	*CpAK1*-qR	TCAGGAAGCCGAGACGGTCA
	*CpAK2*-qF	CGAACGCCTGCCGATTTTGG
	*CpAK2*-qR	ACCGAGGTTGGACGGACAGA
	β-actin-F	AGCGTGAACTGACGGCTCTTG
	β-actin-R	ACTCGTCGTACTCCTGCTTGG
dsRNA synthesis	dsCpAK1-F	TAATACGACTCACTATAGGGAGAAGGAGGTGTTCGACGCTCTG
	dsCpAK1-R	TAATACGACTCACTATAGGGAGACCTTCTCCATGCCGGTCAGC
	dsCpAK2-F	TAATACGACTCACTATAGGGAGAGGAGTTCGAGCGAAGTGGCG
	dsCpAK2-R	TAATACGACTCACTATAGGGAGATCGTAGTGGTCCATCGTCAT
	dsEGFP-F	TAATACGACTCACTATAGGGCCTCGTGACCACCCTGACCTAC
	dsEGFP-R	TAATACGACTCACTATAGGGCACCTTGATGCCGTTCTTCTGC

### Real-time quantitative PCR (RT-qPCR)

RT-qPCR reactions were performed in a CFX 96 Real Time PCR System (Bio-Rad) with SYBR Prime Script RT-PCR Kit II (Takara, Dalian, China) and gene specific primers (Table 1). Before gene expression analysis, the efficiency of primer pairs was verified. The *Cx*. *pipiens pallens β-actin* gene was used as an internal reference gene [12]. Relative gene expressions were calculated using the 2^−ΔΔCT^ method and normalized to the *β-actin* gene in the same sample [13]. The reaction mixture (20μL) contained 10μL of 2×SYBR Premix Ex Taq, 0.8μL of forward primers, 0.8μL of reverse primers, 2μL of template cDNA and 6.4μL of ddH_2_O. All experiments were performed in triplicate and each biological replicate included three technical repeats.

### Sequence analysis

DNAMAN software was used to analyze the sequences of the ORF and noncoding regions of each gene. ClustalW [14] was used for multiple sequence alignments, and MEGA7 was performed to construct phylogenetic tree using the neighbor joining (NJ) method.

### RNA interference (RNAi)

Double-stranded RNAs(dsRNAs) targeting *CpAK1*(dsCpAK1) and *CpAK2*(dsCpAK2) were synthesized using TranscriptAid T7 High Yield Transcription Kit (Thermo Fisher Scientific, Waltham, MA, USA). A total of 200nL of dsRNA was injected into 3-day-old female mosquito. Each mosquito was injected with 200 ng of dsCpAK1, dsCpAK2 or a total of 400 ng of the dsCpAK1 and dsCpAK2 mixture. On the 1st day to 4th day post dsRNA injection, RT-qPCR was used to detect the mRNA expressions of *CpAK1* and *CpAK2*. The dsEGFP-injected female mosquitoes (dsEGFP group) and the uninjected wild-type female mosquitoes (WT group) were set as controls in all injection experiments. All experiments were performed in three independent replicates and each replicate included 30 female mosquitoes.

### Reproduction test

The newly emerged female and male mosquitoes were collected and mixed in the ratio of 1:1.5, put into the cage and fed with 5% sucrose water. Three days later, female mosquitoes were collected and subject to RNAi. The dsEGFP group, dsCpAK1 group, dsCpAK2 group and co-injection of dsCpAK1 and dsCpAK2 group (dsCpAK1+dsCpAK2) were performed for RNAi. Three replications were carried out for RNAi with at least 60 female mosquitoes for dsCpAK1 or dsCpAK2 injection and 120 female mosquitoes for dsCpAK1 and dsCpAK2 co-injection in each replication. After dsRNA injection, mosquitoes in each experimental group were reared separately and fed fresh mouse blood. Survival rate was recorded after 48 hours. Mosquitoes showing no sign of movement when gently touched with a brush were recorded as dead. The numbers of egg rafts laid by surviving mosquitoes, the number of eggs contained in each egg raft and the number of hatched larvae in each egg raft were recorded. Three replications were carried out for reproduction test with at least 30 female mosquitoes in each control or treatment.

### Blood-feeding behavior test

RNAi procedure was the same as reproduction test. At one day after injection, the blood-feeding experiments were performed at three hours before scotophase. The surviving adult female mosquitoes in each RNAi experimental group as described above were observed for blood-feeding behavior. The mice were fixed with clips and placed into cages. Female mosquitoes in each cage were collected at two hours after blood-feeding with an aspirator, and were frozen at -20°C for five minutes. The numbers of blood-feeding and non-blood-feeding female mosquitoes were counted to calculate the blood-feeding rate. To calculate the engorgement rate, the blood-engorged mosquitoes were counted based on the blood feeding-triggered abdominal distension (abdominal blood volume exceeding 1/2 of the whole abdomen). For each treatment, three biological replications were performed under normal feeding conditions.

### Statistical analysis

SPSS 25.0 and GraphPad Prism 6.0 software were used for statistical. All values were presented as the mean ± standard error of the mean (SE). Student’s t-test was used for comparing two means and ANOVA with post-hoc Tukey’s HSD test was used for multiple comparisons of parametric data. Differences were considered statistically significant when *P<0.05 or **P< 0.01. All experiments were conducted in at least three independent replicates.

### Database entries

The cDNA sequences of *CpAK1* and *CpAK2* have been deposited in the GenBank and the accession numbers are MZ491206 and MZ491207, respectively.

## Results

### 2.1 cDNA cloning and sequence analysis of *CpAK1* and *CpAK2*

**Fig 1:**
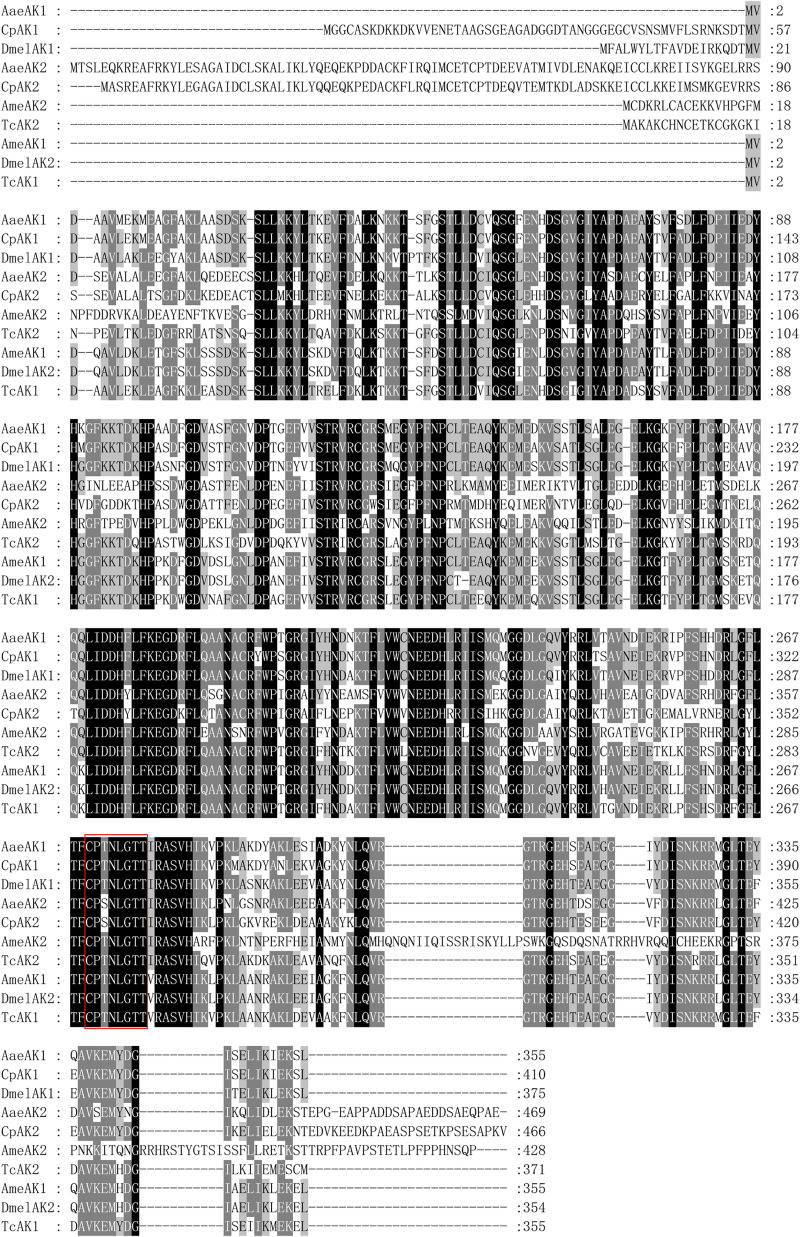
Comparison of amino acid sequences of *CpAK1* and *CpAK2* with other insects including *Aedes aegypti* (*AaeAK1*, *AaeAK2*), *Drosophila melanogaster*(*DmelAK1*, *DmelAK2*), *Apis mellifera* (*AmeAK1*, *AmeAK2*), *Tribolium castanuem* (*TcAK1*, *TcAK2*).

**Fig 2:**
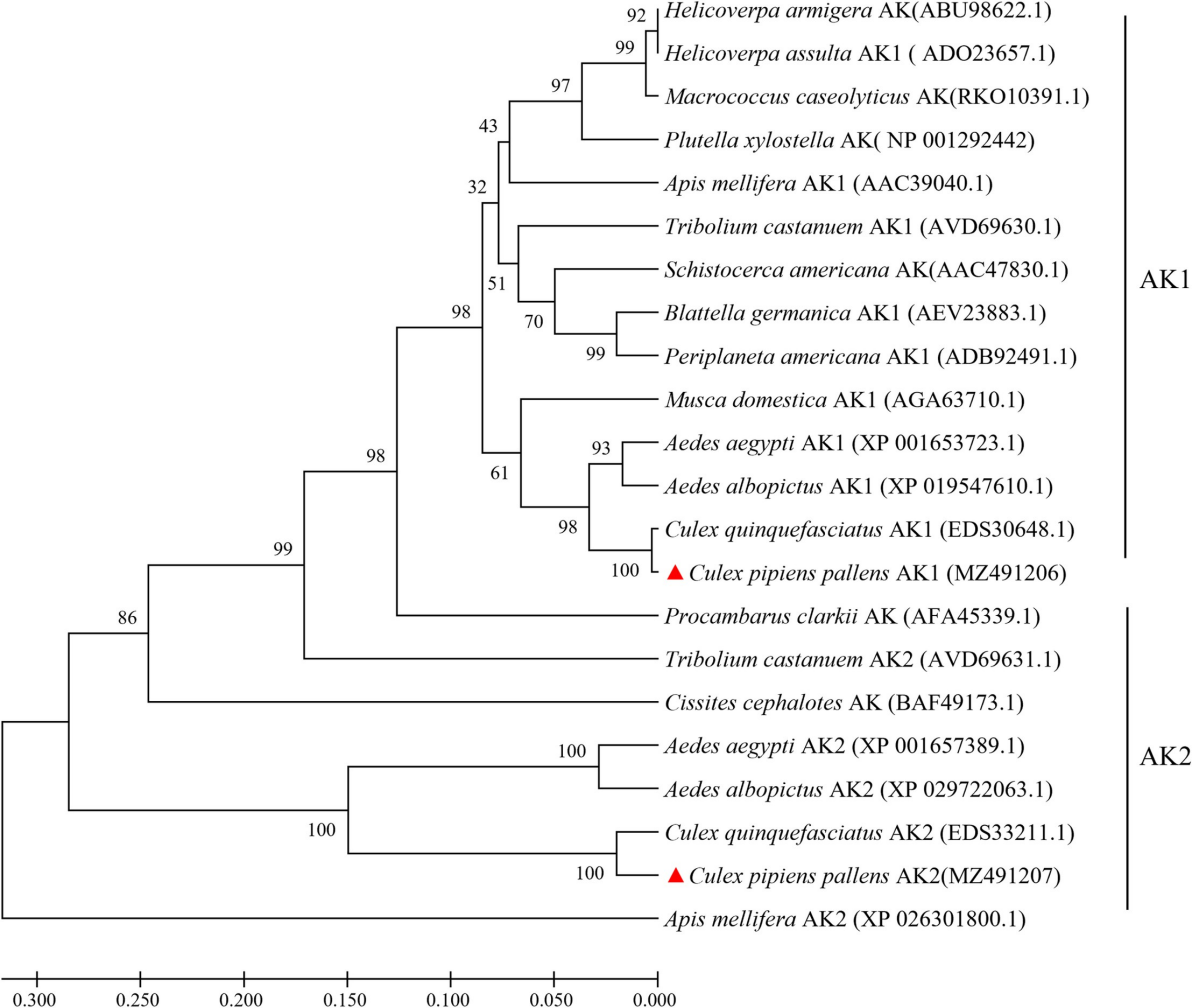
Phylogenetic analysis of arginine kinase genes from *Cx*. *pipiens pallens* and other insects. The phylogenetic tree was constructed using MEGA7 with a boot strapping of 1000 iterations, and an arginine kinase gene (RKO10391.1) from *Macrococcus caseolyticus* was used as an outgroup.

RT-PCR and RACE were used to amplify the entire coding sequences of *CpAK1* and *CpAK2*. The full length *CpAK1* cDNA comprises 1454 bp with 126 bp 5′-untranslated region (UTR), 1233 bp ORF encoding 410 amino acid residues, and 95 bp 3’-UTR. The full length1764 bp *CpAK2* cDNA contains 134 bp 5′-UTR, 1401 bp ORF encoding 466 amino acids, and 229 bp 3′-UTR. The polyadenylation signal (AATAAA) was located 176 bp downstream of the stop codon. Multiple sequence alignments revealed 64.76% amino acid identity between *CpAK1* and *CpAK2*. *CpAK1* and *CpAK2* showed high homology with AK of other insect species, and the CP(S/T)N(I/L)GT signature sequence pattern of ATP-guanidino kinases [15] was conserved in all insect arginine kinases (Fig 1). The phylogenetic analysis showed that CpAK1 and CpAK2 were clustered with insect AK1 and AK2, respectively (Fig 2).

### 2.2 mRNA expression patterns of *CpAK1* and *CpAK2*

**Fig 3:**
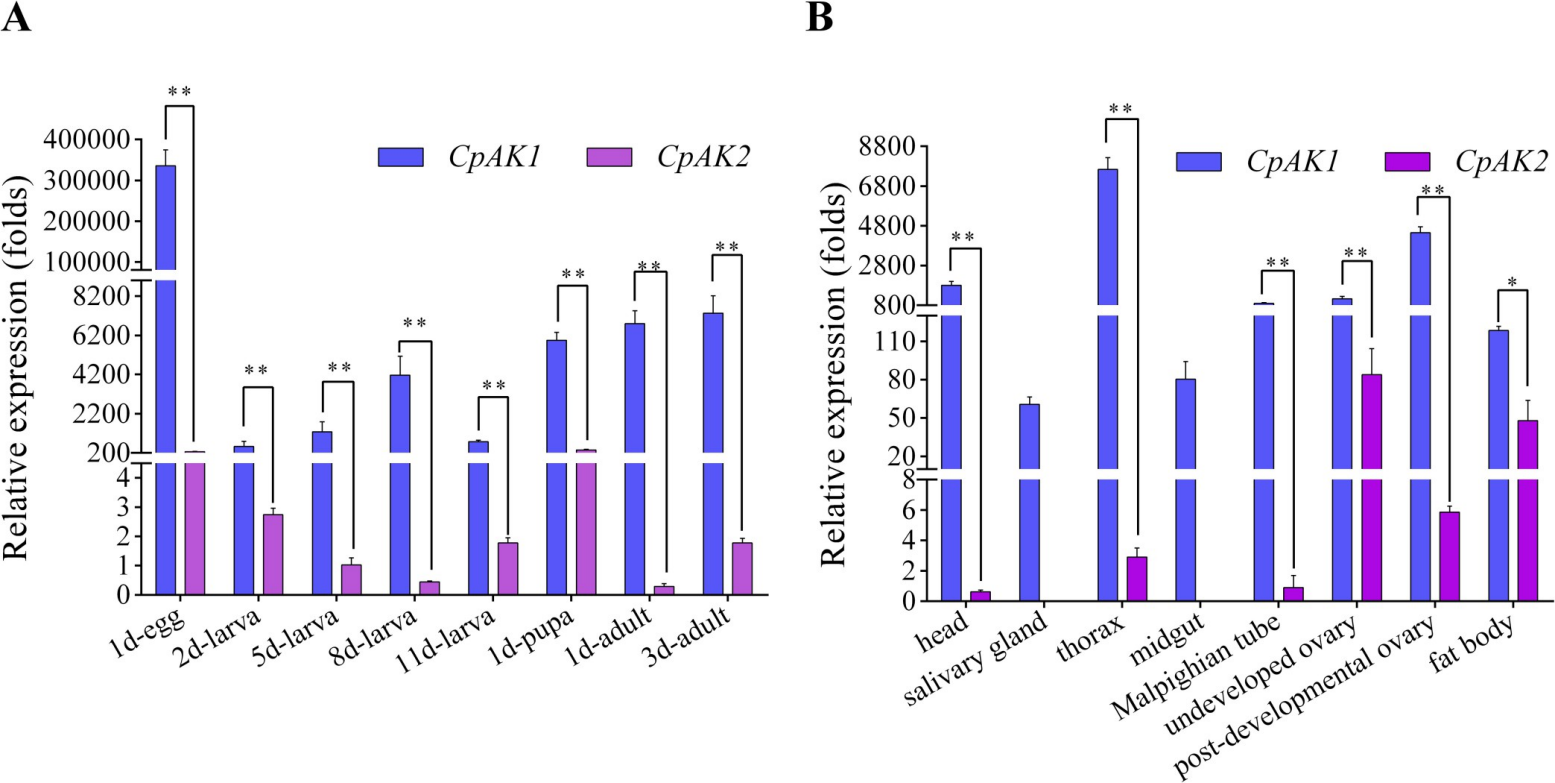
The relative mRNA expression levels of *CpAK1* and *CpAK2* in different developmental stages (A) and various tissues (B) of *Cx*. *pipiens pallen*s. The mRNA levels of target gene were normalized to the reference gene (*β-actin*) in the same sample and calculated using the 2^-ΔΔCT^ method. 1d-egg: 1-day-old egg; 2d-larva: 2-day-old larva; 5d-larva: 5-day-old larva; 8d-larva: 8-day-old larva; 11d-larva: 11-day-old larva; 1d-pupa: 1-day-old pupa; 1d-adult: 1-day-old female adult; 3d-adult: 3-day-old female adult. All data were expressed as the mean ± Standard Error (SE). Significant difference was analyzed by one-way ANOVA test (*P < 0.05, ** *P* < 0.01).

The expression levels of *CpAK1* and *CpAK2* genes in different development stages and different tissues were determined by RT-qPCR. The results showed that *CpAK1* and *CpAK2* were detected in all developmental stages. The relative expression of *CpAK1* was significantly higher than that of *CpAK2* in all examined stages and tissues. *CpAK1* and *CpAK2* showed the highest expression level in eggs and three-day-old adults, respectively. Analysis of the spatial expression profiles of *CpAK1* and *CpAK2* in 3-day-old female adults revealed that *CpAK1* was highly expressed in thorax and post-developmental ovary, whereas the highest expression level of *CpAK2* was observed in undeveloped ovary (Fig 3).

### 2.3 RNAi of *CpAK1* and *CpAK2*

**Fig 4:**
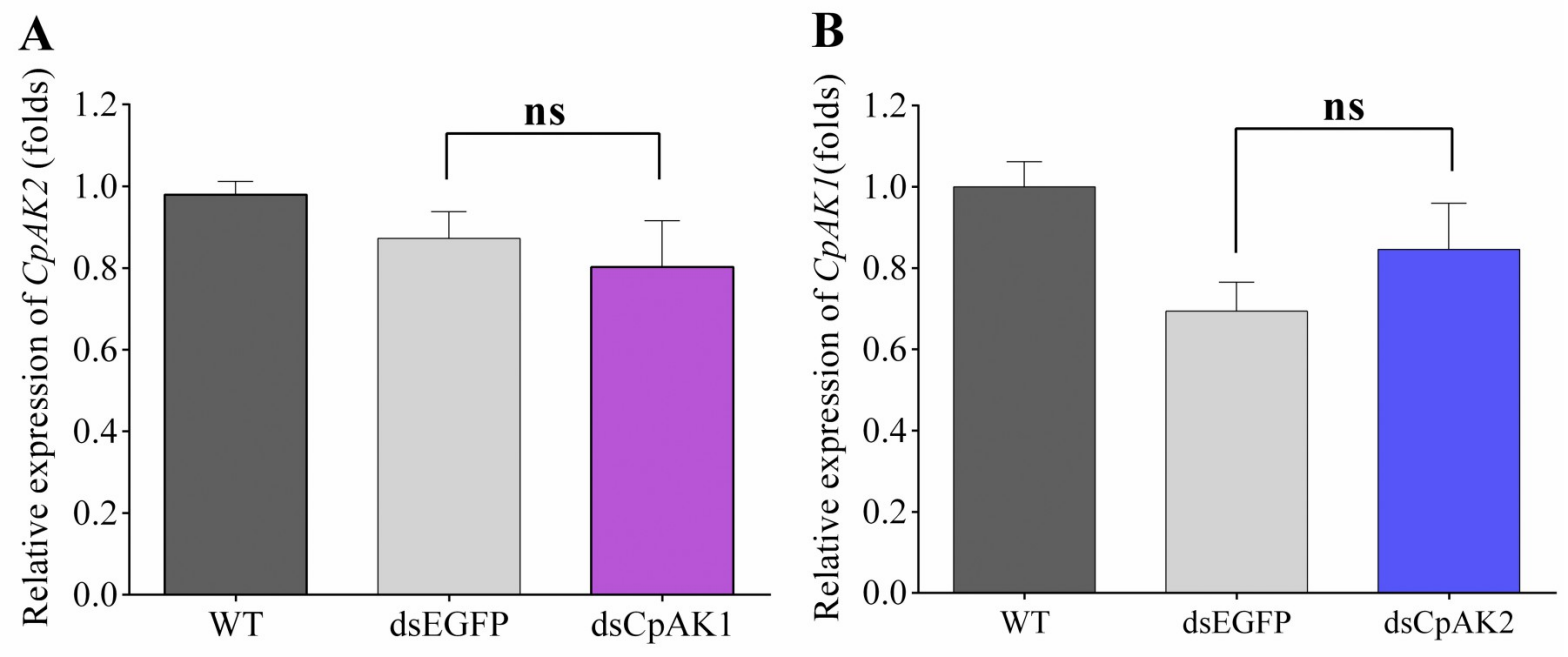
Relative expression levels of CpAK2 (A) and CpAK1 (B) after injection of dsRNAs in *Cx*. *pipiens pallens*. All data were expressed as the mean ± Standard Error (SE).“ns”means “no significant difference”.

**Fig 5:**
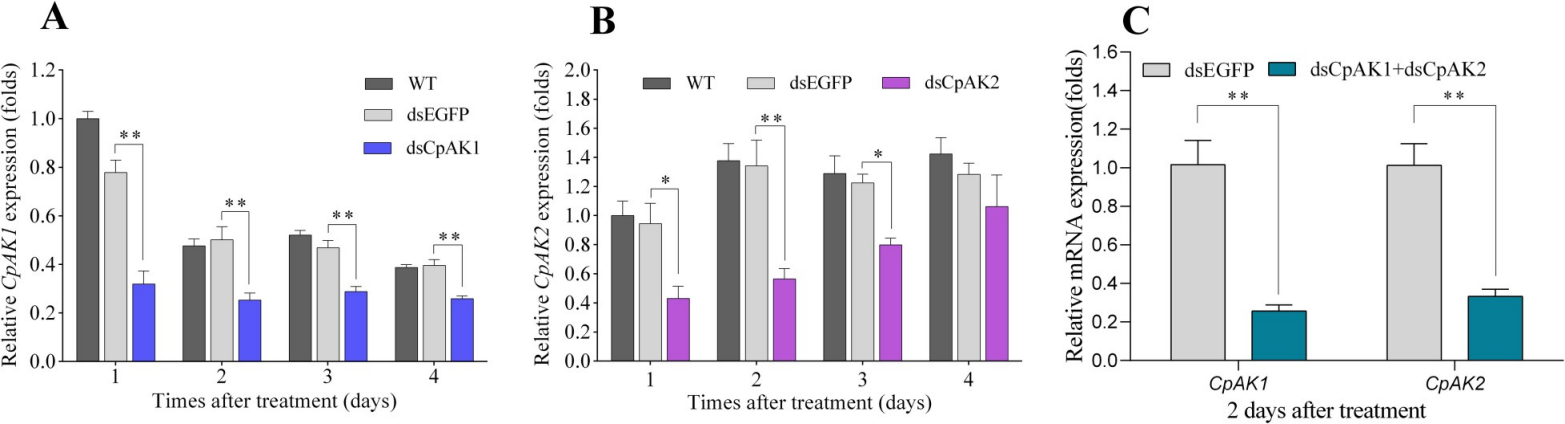
Relative expression levels of *CpAK1* and *CpAK2* after injection of dsCpAK1(A), dsCpAK2(B) and dsCpAK1+dsCpAK2(C) in *Cx*. *pipiens pallens*. All data were expressed as the mean ± Standard Error (SE). Significant difference was analyzed by one-way ANOVA test (*P < 0.05, ** *P* < 0.01).

Considering the relatively high sequence identity between *CpAK1* and *CpAK2*, we first tested whether the two genes interfere with each other. As shown in Fig 4, no cross-interference effect was detected after injection of dsCpAK1 or dsCpAK2 into adults. Treatment with dsCpAK1 led to the reduction of mRNA levels of *CpAK1* by 59.0%, 49.7%, 38.6%, and 34.6% at 1, 2, 3 and 4 days after injection (Fig 5A). The highest RNAi silencing efficiency of *CpAK2* (57.9%) was observed at 48 h post injection (Fig 5B). The suppression efficiencies of *CpAK1* and *CpAK2* were 74.33% and 66.69%, respectively, at two days after co-injection of dsCpAK1 and dsCpAK2 (Fig 5C).

### 2.4 Role of *CpAK1* and *CpAK2* in reproduction

**Fig 6:**
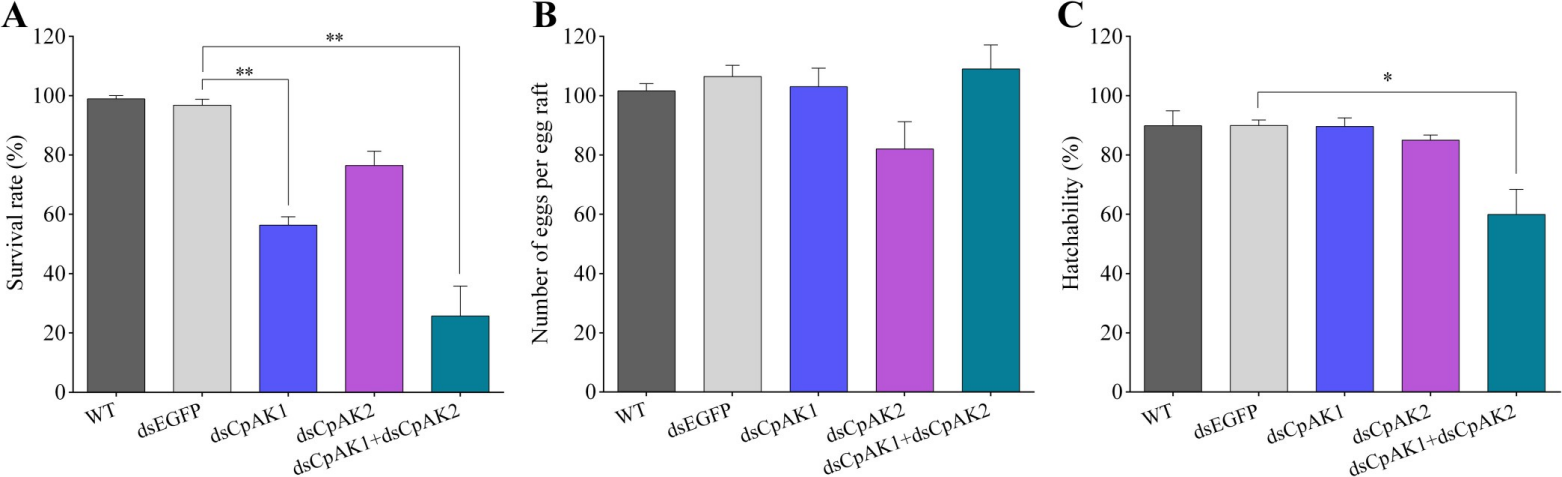
Survival rates (A) of different treatments at 48h after injection. Number of eggs per egg raft (B) and hatchability (C) of female mosquitoes in different treatment groups. All data were expressed as the mean ± Standard Error (SE). Significant difference was analyzed by one-way ANOVA test (*P < 0.05, ** *P* < 0.01).

The effects of *CpAK1 and CpAK2* on fecundity and fertility were investigated after injection of dsRNA into 3-day-old female adults. The survival rates of female mosquitoes at two days after injection of dsCpAK1 and dsCpAK2 were 56.4% and 76.5%, respectively, whereas co-injection of dsCpAK1 and dsCpAK2 led to 25.7% survival rate of mosquitoes (Fig 6A). The female mosquitoes were given a 24 h blood meal at one day after the injection, and the numbers of eggs per egg raft and hatchability were calculated. The results showed that the mean numbers of eggs laid by mosquitoes from dsCpAK1 injection group as well as the dsCpAK1 and dsCpAK2 co*-*injection group were comparable to that from control groups, while the dsCpAK2-injected mosquitoes laid 21.3% fewer eggs per egg raft than control groups (Fig 6B). The hatchabilities of dsCpAK1 group and dsCpAK2 group were not changed significantly when compared with the control groups, whereas the hatchability of dsCpAK1 and dsCpAK2 co*-*injection group were significantly decreased (59.9%) (Fig 6C).

### 2.5 Role of *CpAK1* and *CpAK2* in blood-feeding behavior

**Fig 7:**
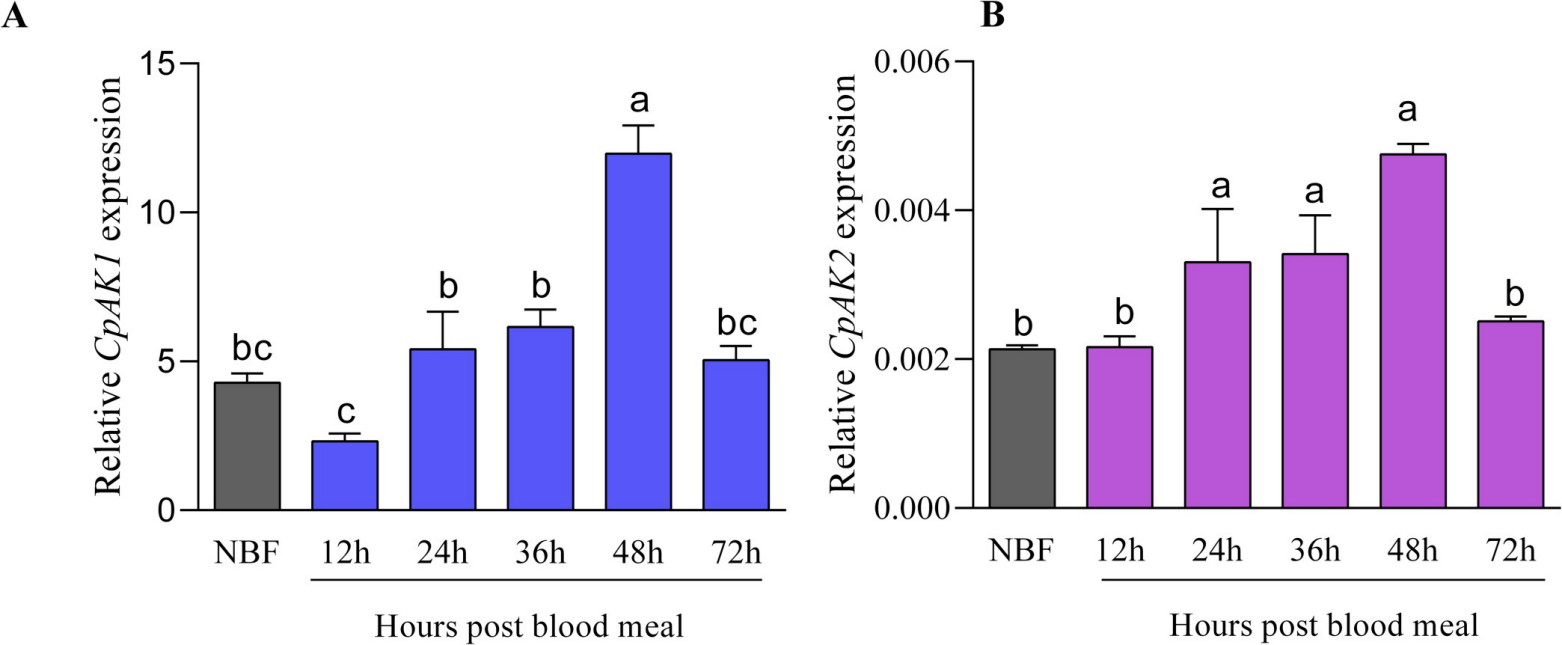
Relative expression of *CpAK1*(A) and *CpAK2*(B) before and after blood feeding. All data were expressed as the mean ± Standard Error (SE). Different letters indicate significant differences among the treatment of the same time point based on the one-way ANOVA test followed by Fisher’s LSD multiple comparison test (*P* ≤ 0.05). “NBF”means “non-blood-feeding”.

The total RNA was extracted from female mosquitoes at different time after blood feeding, and the relative expressions of *CpAK1* and *CpAK2* were detected. Adult female mosquitoes without feeding blood were used as control. The results showed that the mRNA expression levels of both *CpAK1* and *CpAK2* in mosquitoes reached the peak at 48 hours post blood meal, which were significant higher than that in non-blood-feeding mosquitoes, respectively (Fig 7).

**Fig 8:**
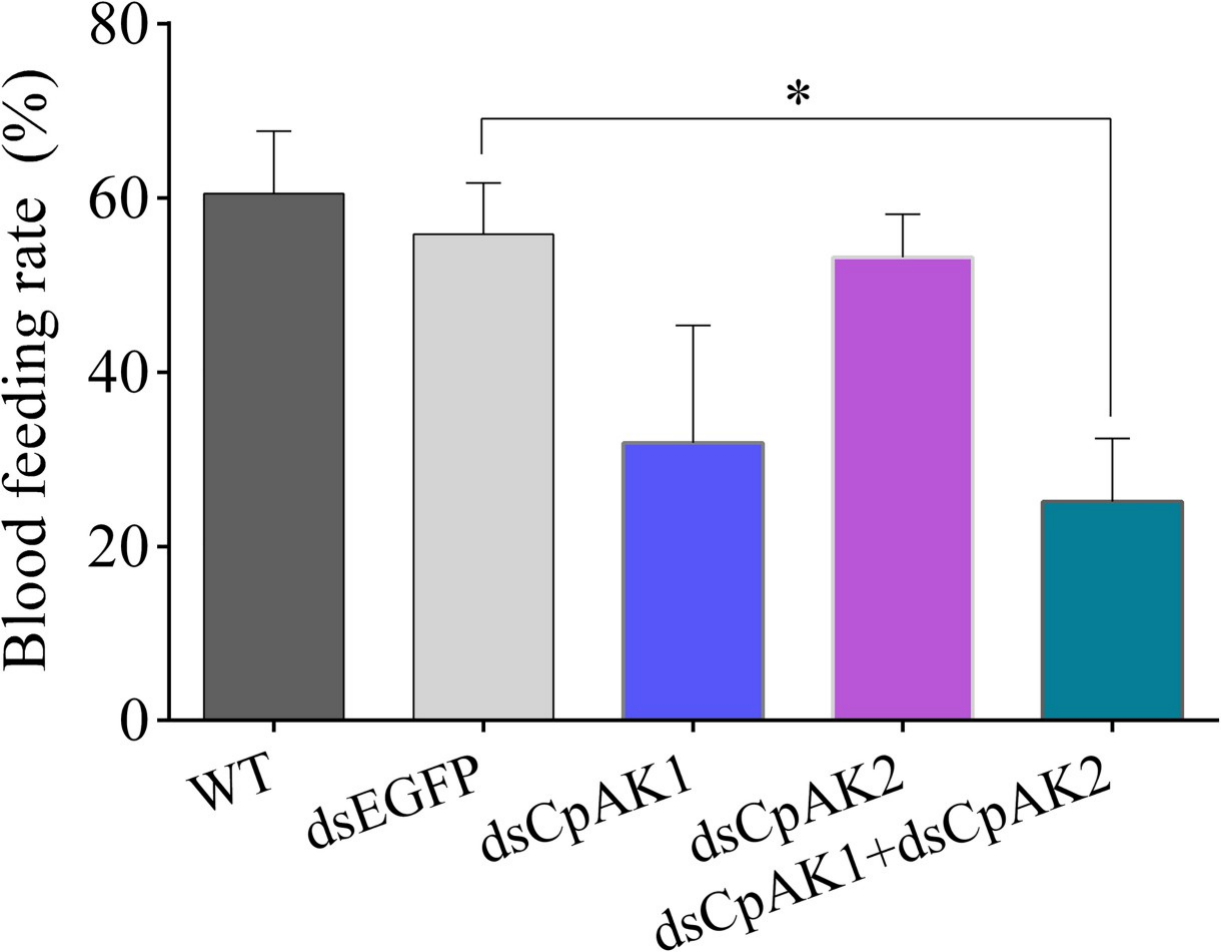
Blood-feeding rate of female mosquitoes in different treatment groups. All data were expressed as the mean ± Standard Error (SE). Significant difference by one-way ANOVA test (*P<0.05).

**Fig 9:**
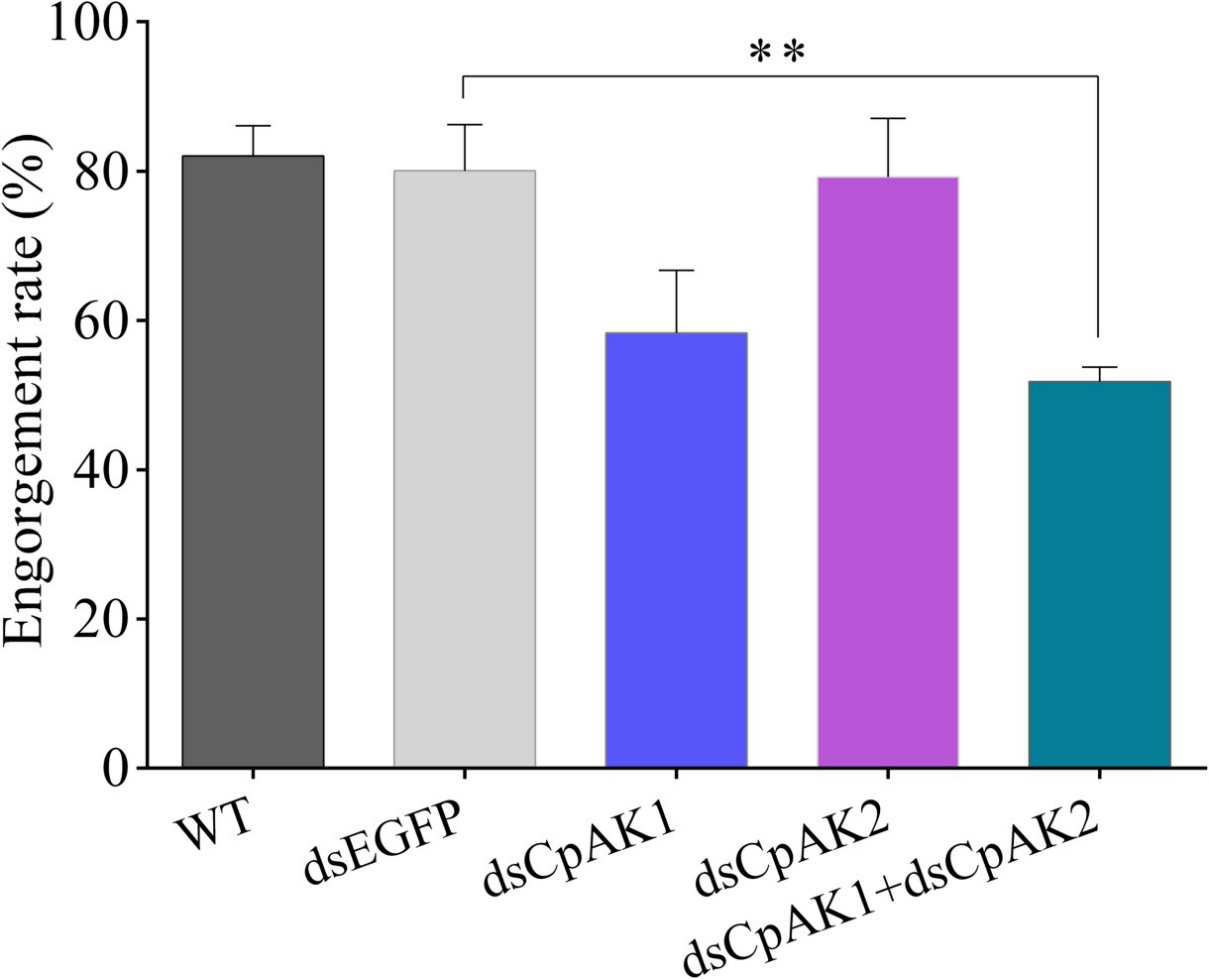
The engorgement rate of female mosquitoes in different treatment groups. All data were expressed as the mean ± Standard Error (SE). Significant difference was analyzed by one-way ANOVA test (** *P* < 0.01).

The role of *CpAK1* and CpAK2 in blood feeding behavior was studied by RNAi. The results showed that dsCpAK2 treatment had no effect on blood feeding behavior of mosquitoes, while the blood feeding rates of female mosquitoes in dsCpAK1 group as well as the dsCpAK1 and dsCpAK2 co*-*injection group were decreased by 28.7% and 35.4%, respectively (Fig 8). The dsCpAK1 treatment slightly decreased the engorgement rate of mosquitoes, whilst the engorgement rate of mosquitoes was significantly decreased by 36.8% after treatment with dsCpAK1 and dsCpAK2 (Fig 9).

## Discussion

Bioinformatic analysis revealed that insect AKs can be divided into two groups [16,17]. Group 1 consists of typical AKs from most insects. Group 2 includes AK2s in *Anopheles gambiae*,*Aedes aegypti*,*Apis mellifera* [18]. While AK genes have been recently cloned from many insects including *Bombyx mori* (L.) [19], *Heliothis assulta* [20], *Blattella germanica* [21], *Nephotettix cincticeps* [22], *Musca domestica* L. [5], *Tetranychus cinnabarinus* [23], *Frankliniella occidentalis* [24], *Tribolium castaneum* [25], comparative characterization of group 1 and group 2 AK genes was only reported in *T*. *castaneum* [25]. In this study, we cloned *CpAK1* and *CpAK2* from *Cx*. *pipiens pallens*. Sequence analysis showed that *CpAK1* and *CpAK2* had characteristic signature sequence pattern of ATP-guanidino kinases. Phylogenetic analysis revealed that *CpAK1* and *CpAK2* was clustered intogroup 1 and group 2 AK of insects, respectively. To our knowledge, this is the first report of molecular characterization of two phylogenetically distant AK genes in mosquitoes.

Arginine kinase system plays the role of ATP buffer in the development stage or tissue with high energy consumption and large fluctuation of energy demand, so the expression levels of arginine kinase genes vary greatly with different development stages and tissues. For example, arginine kinase was expressed at a high level in the brain of *Calliphora erythrocephala* [26]. High expression level of arginine kinase was detected in the body wall and digestive tract of the 3rd instar larvae of *Drosophila melanogaster* [27]. The heads of female and worker ants of *Solenopsis invicta* and the thorax of worker ants exhibited the highest expression level of arginine kinase [28]. High levels of arginine kinase were also found in the midgut of the 5th instar larvae of *Helicoverpa armigera* [6]. In *T*. *castaneum*, the expression level of *TcAK1* in 5th instar larvae and *TcAK2* in 1-day-old males was the highest, and both *TcAK1* and *TcAK2* were highly expressed in the head of 7-day-old female adults [25]. In this study, we found that *CpAK1* and *CpAK2* were highly expressed in egg stage and pupal stage of *Cx*. *pipiens pallen*s. This may be related to the large amount of energy required for the morphological changes of mosquitoes in these two stages. The analysis of spatial expression pattern revealed that the expression of *CpAK1* in the thorax was the highest, followed by the developing ovary. The expression of *CpAK2* was the highest in the undeveloped ovary. The high expression of *CpAK1* and *CpAK2* in ovary indicated their putative role in reproduction.

As a reverse genetics tool, RNA interference has been widely used in the study of insect gene function, including AK. It has been reported that exposure of first instar larvae of *H*. *armigera* to transgenic *Arabidopsis thaliana* expressing dsRNA of *Haak* led to 55% mortality rate and significantly retarded larval growth [8]. Similarly, ingestion of *Lycopersicon esculentum* leaves expressing the dsRNA of *Tuta absoluts* arginine kinase resulted in increased larvae mortality of *T*. *absoluts* [29]. A recent study found that knockdown of *PxAK* in *Plutella Xylostella* prolonged the development time, decreased the weight of pupae and pupation rate, and increased the mortality rate [30]. In this study, we also found that the survival rate of mosquitoes in the dsCpAK1 treatment group decreased significantly, and co-injection of dsCpAK1 and dsCpAK2 led to only 25.7% survival rate. Furthermore, while the average egg number and hatching rate of egg raft did not change significantly after interfering with *CpAK1* and *CpAK2* alone, the hatching rate decreased significantly after co-knockdown of *CpAK1* and *CpAK2*, suggesting synergistic effect of *CpAK1* and *CpAK2* on the reproduction of *Cx*. *pipiens pallens*. Interestingly, we found that the expressions of *CpAK1* and *CpAK2* were significantly up-regulated after blood feeding of *Cx*. *pipiens pallens*. Further RNAi experiment revealed that co-injection of dsCpAK1 and dsCpAK2 significantly decreased the blood feeding rate and the engorgement rate of female mosquitoes. These results suggested that as the crucial enzyme involved in energy metabolism, AK plays an important role in the blood feeding, a highly energy-demanding process in mosquitoes. Since blood feeding is critical for mosquito reproduction and disease transmission [31], RNAi-mediated knockdown of AK might provide a novel control strategy of mosquito born disease.
